# Age-dependent association of gut bacteria with coronary atherosclerosis: Tampere Sudden Death Study

**DOI:** 10.1371/journal.pone.0221345

**Published:** 2019-08-22

**Authors:** Sari Tuomisto, Heini Huhtala, Mika Martiskainen, Sirkka Goebeler, Terho Lehtimäki, Pekka J. Karhunen

**Affiliations:** 1 Finnish Cardiovascular Research Center Tampere, Faculty of Medicine and Health Technology, Tampere University, Tampere, Finland; 2 Fimlab Laboratories Ltd, Pirkanmaa Hospital District, Tampere, Finland; 3 Faculty of Social Sciences, Tampere University, Tampere, Finland; 4 National Institute for Health and Welfare, Tampere, Finland; University of Illinois at Urbana-Champaign, UNITED STATES

## Abstract

**Background:**

The gut microbiome is thought to remain stable into old age. Gut bacteria and their translocation may play a role in the development of coronary heart disease (CHD) by modulating cholesterol levels and immune responses, as well as by producing toxic metabolites and bacterial endotoxins. The association of changes in the gut microbiome with the severity of coronary atherosclerosis and the ability of gut bacteria themselves to translocate into coronary plaques has not been studied.

**Materials and methods:**

As a part of the Tampere Sudden Death Study, we measured age-dependent changes in the relative ratios of major intestinal bacterial communities (*Bacteroides* species [spp.], the *Clostridium leptum* group, the *Clostridium coccoides* group, *Bifidobacterium* spp., *Enterobactericeae*, *Lactobacillus* spp.) and *Streptococcus* spp. in both feces and coronary plaques of the same male autopsy cases (n = 67, age range 44–95) using real-time quantitative PCR (qPCR). The area of coronary atherosclerotic lesions were measured by computer-assisted morphometry. Fecal bacterial DNA measurements from healthy volunteers served as a control for gut bacterial analyses of autopsy cases. The relative amount of bacterial DNA in a sample was determined with the comparative Cq method.

**Results:**

The relative ratios of fecal *Lactobacillus* spp., *Bifidobacterium* spp., the *Clostridium coccoides* group, and *Bacteroides* spp. did not differ between controls and autopsy cases and showed no age-dependence. In contrast, the ratios of the *Clostridium leptum* group, *Enterobactericeae*, and *Streptococcus* spp. increased with age. Elevated relative ratios of fecal *Enterobactericeae* associated with a larger coronary plaque fibrotic area (*p = 0*.*001*), and the *Clostridium leptum* group with a larger calcification area (*p = 0*.*015*). Intestinal bacterial DNA could be amplified in 67.6% of the coronary plaques, the most common being *Streptococcus* spp. (41.0%), followed by *Enterobactericeae* (12.1%), *Clostridium leptum* (2.4%), and *Lactobacillus* spp. (2.4%). The percentages of *Streptococcus* spp. DNA decreased, and those of *Enterobactericeae* increased in coronary plaques along with age.

**Conclusions:**

DNA of the *Clostridium leptum* group and pathogenic *Enterobactericeae* increase in the gut microbiome with age and can be detected in the same individual’s coronary plaques along with pathogenic *Streptococcus* spp., associating with more severe coronary atherosclerosis.

## Introduction

Coronary heart disease (CHD) is one of the leading causes of death worldwide. Many CHD risk factors, such as smoking, hypertension, poor diet, dyslipidemia, lack of exercise, obesity, adiposity, and diabetes mellitus, are associated with lifestyle and therefore modifiable. It has long been speculated that gut bacteria might have a role in the development of CHD by modulating several signaling pathways in the host, such as lipid metabolism and inflammation [1]. Moreover, the DNA of several bacteria that originate from the gastrointestinal tract—such as *Proteobacteria* (*e*.*g*. *Enterobacter* spp.), *Cryseomonas* spp., *Veillonella* spp., *Streptococcus* spp., *Staphylococcus* spp., *Propionibacterium* spp. [2], and *Chlamydia* spp. [3]—have been found in coronary plaques. This suggests that the translocation of bacteria or their residuals, such as endotoxins, from the intestine might be considered a possible mechanism for chronic plaque inflammation. Bacterial translocation from the mouth or other parts of the gastrointestinal tract into circulation is common after dental or surgical procedures, endoscopy, manipulation, or local mucosal infections. Physiological processes, such as defecation, can also lead to a transient detection of bacterial material in the circulation [3]. Furthermore, in patients with CHD, the structure and permeability of the intestinal epithelium has been shown to be altered [4], enhancing the possibility of bacteria and/or their residuals translocating from the gut into the epithelium and continue into blood.

The main beneficial functions of normal intestinal bacteria populations include protecting the host against invading pathogens (known as colonization resistance), supporting energy metabolism by digesting carbohydrates and proteins that the host cannot digest on its own, modulating the function and structure of the immune system, as well as vitamin (vitamin K and some B-vitamins) biosynthesis and mediating the breakdown of dietary carcinogens [5, 6]. The most dominant taxa normally present in the intestine are *Bacteroidetes*, *Firmicutes*, *Proteobacteria*, and *Actinobacteria* [7]. *Bacteroidetes* and *Firmicutes* phyla are the majority, representing 56% and 29% of the microbiota, respectively, followed by *Actinobacteria* at 6% and *Proteobacteri*a at 4% [8]. At the species level, a healthy individual’s gut microbiota mainly consists of *Bacteroides* spp., *Bifidobacterium* spp., *Enterobacte*r spp., *Peptostreptococcus* spp., and bacteria belonging to the *Clostridium coccoides* (cluster XIVa) and *Clostridium leptum* (cluster IV) groups [9, 10]. Fecal *Streptococcus* spp. have been reported to represent 1%–10% of the total aerobic intestinal flora and, together with *Enterobactericeae*, comprise the main facultative anaerobes [11]. *Lactobacillus* spp. has been estimated to present ≤1% of the total bacterial population in the distal human gut and is estimated to constitute approximately 0.3% of all bacteria in the colon [12].

During the first three years of life, the intestinal microbial community becomes stable [13]. After this it is mainly modified by diet, bacterial infections, antibiotics, surgeries, or other major invasions [14, 13]. In old age there is a decline in microbiota diversity. According to Pérez Martínez et al. and Rondanelli et al., there is an increase in *Proteobacteria*, a decrease in Bifidobacteria, and a decrease in the ratio of *Firmicutes* to *Bacteroidetes* [15, 16]. Age-related perturbation in the gut microbiome is suggested to be an important determinant of age-associated pathological states, such as chronic inflammation [17] and cardiovascular disease [18], possibly due to a decline in immune system function—*e*.*g*. immunosenescence [19]. Such a change in the functional profile may be associated with an increase in the proportion of pathogenic bacteria commonly present in low numbers in the adult gut ecosystem. However, it is not known whether changes in the normal gut microbiome composition might associate with the severity of coronary atherosclerosis.

The aim of this study was to investigate age-dependent changes in the major populations of the intestinal microbiome and their possible association with atherosclerotic severity and death due to CHD. Furthermore, we studied whether such intestinal derived bacterial DNA can be found in coronary plaques, which could indicate the translocation of gut bacteria via the portal vein into circulation and further into coronary plaques. Six groups, namely *Bacteroides* spp. (*Bacteroidetes*), *Clostridium* spp. (*Firmicutes*), *Streptococcus* spp. (*Firmicutes*), *Lactobacillus* spp. (*Firmicutes*), *Bifidobacterium* spp. (*Actinobacteria*), and *Enterobactericeae* (*Proteobacteria*), were chosen, since they represent the genera of the major phyla [20].

## Materials and methods

The present study comprises a prospective series of 67 males (mean age 59 years, range 18–95 years, Table 1) subjected to medico-legal autopsy in the Department of Forensic Medicine at the University of Tampere as a part of the Tampere Sudden Death Study. The selection criteria for the cases were: out-of-hospital death, male gender, time elapsed post-mortem less than 6 days, intact middle torso and bowel, no signs of bacterial infections or drug addiction, and no visible wounds or necrosis. Death due to CHD was determined by forensic pathologists according to cause of death selection guidelines by WHO from 1965 as well as the most recent 10^th^ version of the International Classification of Diseases (ICD-10), and based on autopsy findings, microscopic examinations, and toxicological screening. Of the cases, 34 (51%) died of CHD with or without an old myocardial infarction, and 33 (49%) died of non-CHD-related causes. There were no cases with acute myocardial infarction in this series. As expected, CHD cases were significantly older and suffered from more severe coronary atherosclerosis than non-CHD-related death cases. There were no statistical differences in BMI or post-mortem time.

**Table 1:** Demographic characteristics of the study subjects.

	CHD cases	Non-CHD Deaths	Healthy volunteers	P-value
N	34	33	7	
Post mortem time (days, mean)	4	3		0.270
Age mean (range)	68 (44–95)	49 (18–75)	45 (26–57)	0.000
BMI mean (range)	26.8 (20.7–48.0)	28.6 (15.2–42.8)	27.1 (20.8–37.2)	0.409
Cause of death				
CHD %	100 (100%)	0 (0%)		
other disease %	0 (0%)	16 (48%)		
non-natural death^1^	0 (0%)	17 (52%)		
Coronary atherosclerosis				
fatty streak area (%)	7.4 (0–32.6)	7.4 (0–54.7)	-	0.871
fibrotic lesion area (%)	15.8 (0–66.5)	16.6 (0–42.0)	-	0.585
calcification area (%)	20.4 (0–26.5)	3.18 (0–78.2)	-	0.000
Coronary stenosis (%)	58.1 (0–100)	26.8 (0–80.7)	-	0.000

^1^ suicide, accident, poisoning

CHD = coronary heart disease. BMI = body mass index. P-values over the groups were calculated with ANOVA.

The time interval between death and storage of the body in the mortuary was less than 24 hours in all cases. In the mortuary, the bodies were kept at 4°C. Low temperatures prevent bacterial growth, with bacterial populations unlikely to alter. Based on hospital records, incident police reports with data on drugs found at the home and possible treatments, as well as physician admission notes, none of the victims suffering sudden out-of-hospital death used antibiotics within two weeks prior to death.

Fecal samples of 7 healthy volunteers were collected to serve as healthy controls for comparison with the deceased study subjects. The mean age of the volunteers was significantly younger compared to autopsy cases. Healthy volunteers had not used antibiotics prior to sampling.

### Ethics statement

The study was approved by the Ethics Committee of the Pirkanmaa Hospital District and the National Supervisory Authority for Welfare and Health (VALVIRA). Written consent was obtained from volunteers.

### Analysis of relative ratios of gut bacteria in fecal samples

Fecal samples were taken aseptically from the rectum of autopsy cases, transferred to the laboratory in closed sterile Petri dishes, immediately frozen and stored at -80°C until further processing. Bacterial DNA from 150 mg (wet weight) was extracted from samples using a commercial DNA extraction kit (Zymo Fecal DNA Kit, Zymo Research Corporation, Irvine, California, USA) according to the instructions provided.

The relative quantity of major gut bacteria populations in a sample was determined by qPCR using specific oligonucleotide primers and probes (Table 2) for *Bacteroides* spp. [21], the *Clostridium leptum* group [22], the *Clostridium coccoides* group [22], *Bifidobacterium* spp.[22], *Enterobactericeae* [23], *Lactobacillus* spp. [23], and *Streptococcus* spp., mainly of the *Str*. *mitis*-group (recognition of *Str*. *mitis*- group (*Str*. *mitis Str*. *oralis*, *Str*. *gordonii*, *Str*. *sanguinis*, *Str*. *pneumonia*), *Str*. *salivarius*, *Str*. *thermophilus*, uncultured streptococci, *Lactobacillus lactis*) [24], as previously presented [25, 24]. The total amount of gut bacteria was measured using universal bacterial primers and probes as earlier described [26]. Amplification primers and probes were synthesized according to sequences published and verified by BLAST on the National Centre for Biotechnology Information server (http://www.ncbi.nlm.nih.gov/) and Ribosomal Database Project (http://rdp.cme.msu.edu/probematch/search.jsp). The specificity and cross-reactivity of the designed primers and probes were tested using bacterial cultures from clinical samples as earlier described [25, 23, 24]. The results of *Streptococcus*, mainly of the *Str*. *mitis*-group were marked as *Streptococcus* spp..

**Table 2:** The primers and probes used.

Primer and probe	Sequence (5’-3’)	Reference
***Bacteroides* spp.**		
Forward	TGGTAGTCCACACAGTAAACGATGA	**[21]**
Reverse	CGTACTCCCCAGGTGGAATACTT	
Probe	GTTTGCCATATACAGTAAGCGGCCAAGCG	
***Bifidobacterium* spp.**		
Forward	CGGGTGAGTAATGCGTGACC	**[22]**
Reverse	TGATAGGACGCGACCCCA	
Probe	CTCCTGGAAACGGGTG	
***Clostridium leptum* group**		
Forward	CCTTCCGTGCCGSAGTTA	**[22]**
Reverse	GAATTAAACCACATACTCCACTGCTT	
Probe	CACAATAAGTAATCCACC	
***Clostridium coccoides* group**		
Forward	GACGCCGCGTGAAGGA	**[22]**
Reverse	AGCCCCAGCCTTTCACATC	
Probe	CGGTACCTGACTAAGAAG	
***Enterobactericeae***		
Forward	GCGGTAGCACAGAGAGCTT	**[23]**
Reverse	GGCAGTTTCCCAGACATTACTCA	
Probe	CCGCCGCTCGTCACC	
***Streptococcus spp*., mainly the *Str*. *mitis-*group**		
Forward	CCAGCAGCCGCGGTAATA	**[24]**
Reverse	CCTGCGCTCGCTTTACG	
Probe	ACGCTCGGGACCTACG	
***Lactobacillus* spp.**		
Forward	GCTAGGTGTTGGAGGGTTTCC	**[23]**
Reverse	CCAGGCGGAATGCTTAATGC	
Probe	TCAGTGCCGCAGCTAA	
**Universal**		
Forward	TGGAGCATGTGGTTTAATTCGA	**[26]**
Reverse	TGCGGGACTTAACCCAACA	
Probe	CACGAGCTGACGACA[A/G]CCATGCA	

The efficiency of the used universal primers and probe calculated from the dilution curve has been ca. 82%-100% (amplification factor 1.87–2.00), for *Bacteroides* spp. primers and probe 99% (amplification factor 1.99), *Bifidobacterium* spp. 100% (amplification factor 2.00), *C*. *leptum* group 106% (amplification factor 2.06), *C*. *coccoides* group 106% (amplification factor 2.06), *Streptococcus* spp, mainly *S*. *mitis* group 97% (amplification factor 1.97), *Lactobacillus* spp. 99% (amplification factor 1.99), *Enterobactericeae* 97% (amplification factor 1.97), RNaseP 104% (amplification factor 2.04). In the relative qPCR method, the result is given in relation to the reference.

Assays for fecal samples were performed with the AbiPrism 7500 HT Sequence Detection System (Taqman, Applied Biosystems, USA) in a reaction volume of 20 μl in 96-well reaction plates under standard conditions, using specific Taqman allele hybridization according to instructions. The AbiPrism 7900 HT Sequence Detection System (Taqman, Applied Biosystems, USA) was used for coronary artery samples with a reaction volume of 5 μl. All amplifications and detections were carried out as duplicates or quadruplicates (in uncertain cases). The Master Mix was prepared using Taqman Environmental Master Mix for fecal samples, with a final concentration of 1000 nM for each primer, and 250 nM for each fluorescently labeled probe. DNA from fecal samples were diluted to a ratio of 1:100. One microliter of sample DNA was added to PCR reactions for detection.

The amplification data were analyzed with SDS 2.2 software (Applied Biosystems), which calculates ΔRn using the equation Rn(+)−Rn(−). Rn(+) is the emission intensity of the reporter divided by the emission intensity of the quencher at any given time, whereas Rn(−) is the value of Rn(+) prior to PCR amplification. Thus, ΔRn indicates the magnitude of the signal generated. The critical threshold cycle (Cq) is the cycle at which a statistically significant increase in ΔRn is first detected and at which the fluorescence becomes detectable above the background. Cq is inversely proportional to the logarithm of the initial number of template molecules, *i*.*e*. the initial amount of sample DNA.

The relative amount (N-fold value) of bacterial DNA in the sample was determined with a comparative Cq method (ΔΔCq, ΔCq_sample_−ΔCq_reference sample_), applied with a simplification [25, 27, 28, 29, 30]. First, ΔCq differences between specific bacterium and universal bacterium measurements were calculated for each sample, then ΔΔCq for the sample and reference. As a reference value for fecal ΔΔCq calculation for cases of CHD vs. non-CHD deaths, the mean Cq value from the non-CHD autopsy group was used. Similarly, for age-dependent fecal calculations, the mean Cq value from the <50 group was used as a reference. This calculation method yields an n-fold difference in the amounts of specific bacteria between samples and the reference.

### Computer-assisted morphometric measurement of coronary atherosclerosis

Coronary arteries were opened aseptically and stored on closed sterile Petri dishes then fixed onto cardboard with needles for digital computer-assisted morphometric quantification of the plaque (with the program Olympus cell^D, Greece). A transversally cut piece containing the most severe atheroma or plaque of the coronary artery—the predilection site of atherosclerosis—was stained using the Verhoeff-hematoxylin eosin method to visualize the internal and external elastic membranes and allow measurement of coronary stenosis percentage.

### Analysis of coronary plaque bacterial DNA

For DNA extraction, a transversally cut piece from the most severe coronary atheroma of the coronary artery was surgically extracted and stored at -80°C until DNA extraction with the Qiagen MiniKit (Qiagen, Germany). The relative quantity of bacteria in plaque samples was determined by qPCR with universal bacterial primers and specific oligonucleotide primers and probes for intestinal bacteria, as described above, using universal measurements as a reference for ΔCq calculation. Maxima Probe/ROX qPCR Master Mix (Thermo Scientific, Massachusetts, USA) was used for qPCR assays on coronary plaque samples. DNA from coronary samples was not diluted for PCR. For determination of the total amount of bacterial DNA, a commercially available human housekeeping gene, RNaseP (TaqManCopy Number Reference Assay, Applied Biosystems, Foster City, CA), was used as a reference [30, 31, 32].

For coronary plaque samples, a ΔCq value of a combined 4 control cases with healthy arteries (fatty streak, fibrotic lesion, and calcified plaque area all 0%), was used as a reference to determine bacterial DNA positivity and relative amounts in samples. The differences in Cq values between candidate bacteria and universal bacteria measurements (ΔCq) were calculated for each sample, after which a comparative Cq (ΔΔCq) for the sample and reference was calculated. Samples were marked as positive for the candidate bacteria, if 2^-ΔΔCq^ > = 2 [24, 33].

### Statistics

Median values and statistical calculations for the different study groups were calculated using IBM SPSS Statistical Software version 21 (IBM Corp. released 2012. IBM SPSS Statistics for Windows, Version 21.0. Armonk, NY: IBM Corp.). The Kruskal-Wallis median test was used to measure significant differences over the groups in intestinal bacterial samples. If these were less than 0.05, post hoc pairwise comparisons with the Mann-Whitney U test were performed. In the case of less than three different study groups, the Mann-Whitney U test was used. A p-value of less than 0.05 was considered statistically significant. Bonferroni correction to control multiple testing was implemented, the p-value cutoffs defined as 0.05/7 bacterial groups = 0.007. Correlations were calculated with Spearman’s rho.

## Results

### Major commensal Gut bacteria in healthy volunteers and autopsy cases

Median values of relative amounts of the measured bacterial groups in fecal samples of autopsy cases were similar to those of healthy volunteers (1.1 vs. 1.3, p = 0.793, for *Bacteroides* spp.; 1.3 vs. 1.4, p = 0.420, for the *Clostridium leptum* group; 0.8 vs. 1.0, p = 0.822, for the *Clostridium coccoides* group; 1.1 vs. 0.8, p = 0.694, for *Bifidobacterium* spp.; 0.9 vs. 0.8, p = 0.852, for *Enterobactericeae*; 2.6 vs. 1.1, p = 0.486, for *Lactobacillus* spp.; and 3.3 vs. 2.7, p = 0.408, for *Streptococcus* spp. mainly of the *Str*. *mitis* group (Mann-Whitney U test; data not shown)).

### Age-dependent changes in relative ratios of major commensal gut bacterial populations

**Fig 1:**
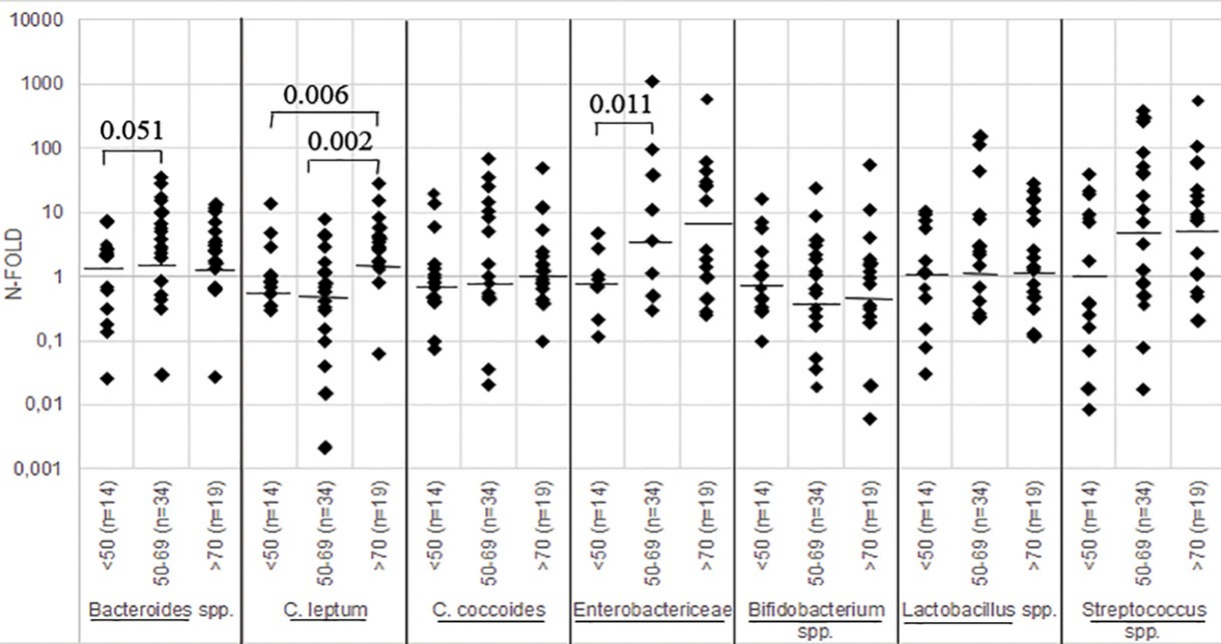
Age-dependent changes in gut bacterial populations in autopsy fecal samples. **Age-dependent changes in relative amounts (*i*.*e*. n-fold differences) of the measured bacteria** (***Bacteroides*** spp., ***Clostridium*** (***C*.*) leptum* group, *C*. *coccoides* group, *Enterobactericeae***, ***Bifidobacterium*** spp., ***Lactobacillus*** spp., ***Streptococcus* spp.).** Individual values are presented as boxes, median values as horizontal lines. As a reference value for fecal ΔΔCq calculation, the mean Cq value from the <50 age group was used. Comparisons were calculated first over the groups with the Kruskall-Wallis test and then pairwise with the non-parametric Mann-Whitney U test.

Inter-individual and within-individual differences were high in all the measured intestinal bacteria in gut samples. The relative N-fold ratio values of *Bacteroides* spp., the *Clostridium coccoides* group, *Bifidobacterium* spp., and *Lactobacillus* spp. did not change with advancing age (Fig 1). However, the relative ratios of the *Clostridium leptum* group (p = 0.003, Kruskal-Wallis), *Enterobactericeae* family (p = 0.056), and *Streptococcus* spp. (n.s.) increased with age: relative ratios of the *Clostridium leptum* group were highest in the oldest age group (>70 years, n = 19), whereas ratios of *Enterobactericeae* were already significantly (p = 0.011) increased in the middle-aged group (50–69 years; n = 34) in comparison to those aged under 50 years (n = 14). Relative N-fold ratios of *Streptococcus* spp. increased with age from 1.1 to 7.4 in middle age and to 7.7 in the oldest age group, but these differences were not statistically significant.

### Major commensal gut bacteria in CHD and non-CHD cases

When comparing amounts of intestinal bacteria in cases of CHD-related death against non-CHD-related deaths, three times more (n-fold 3.4 vs 0.9, p = 0.043) bacteria of the *Clostridium leptum* group were found in victims of out-of-hospital sudden cardiac death. While a decrease in the amount of *Bifidobacterium* spp. (n-fold 1.2 vs 0.6) was also observed, this and other differences were not statistically significant.

### The association of changes in gut bacteria with coronary atherosclerosis

Association of the differences in relative ratios of the measured intestinal bacterial genomes in the gut with the extent and severity of coronary atherosclerosis were then analyzed using values below or above the median fatty streak areas as well as fibrotic plaque and calcified plaque areas (Table 3). Increased ratios of fecal *Enterobactericeae* were associated with a larger coronary plaque fibrotic area (20.96 vs 0.85; p = 0.001) and increased ratios of the *Clostridium leptum* group with a larger calcification area (3.50 vs. 0.89; p = 0.015). Increased ratios of *Streptococcus* spp. in the gut (4.72 vs. 0.59; p = 0.061) also tended to associate with coronary calcification areas.

**Table 3:** The association of gut bacteria with atherosclerotic lesions.

Gut bacteria	Atherosclerosis Lesion Type	Atherosclerosis Area (N-fold differences)		
		<Median	>Median	P-value
***Bacteroides* spp.**	Fat %	1.20	0.89	**0.168**
	Fibrosis %	1.21	1.44	**0.896**
	Calcification %	1.69	2.12	**0.177**
***C*. *leptum* group**	Fat %	1.74	1.00	**0.783**
	Fibrosis %	0.97	1.96	**0.265**
	Calcification %	0.89	3.50	**0.015**
***C*. *coccoides* group**	Fat %	0.51	0.48	**0.635**
	Fibrosis %	0.72	1.22	**0.537**
	Calcification %	0.85	0.64	**0.605**
***Bifidobacterium* spp.**	Fat %	1.23	0.98	**0.778**
	Fibrosis %	1.47	0.49	**0.344**
	Calcification %	0.76	0.63	**0.582**
***Enterobactericeae***	Fat %	0.62	1.00	**0.869**
	Fibrosis %	0.85	20.96	**0.001**
	Calcification %	0.97	0.62	**0.557**
***Lactobacillus* spp.**	Fat %	0.74	0.93	**0.347**
	Fibrosis %	1.14	0.79	**0.812**
	Calcification %	0.96	1.74	**0.145**
***Streptococcus* spp.**	Fat %	2.44	2.14	**0.537**
	Fibrosis %	2.22	2.02	**0.865**
	Calcification %	0.59	4.72	**0.061**

Association of the relative amounts (n-fold differences) of measured intestinal bacteria in fecal samples with extent of atherosclerotic lesions (% of coronary surface area) in left ascending coronary artery (LAD) coronary plaques. The fatty streak area and areas of fibrotic and calcified lesions were dichotomized into below-median (n = 33) and above-median (n = 33) groups (the median value was 3.03 for fat, 13.45 for fibrosis, and 3.93 for calcification). N-fold values for bacteria were calculated using the average Cq value in the <median group as a reference. P-values have been calculated with the non-parametric Mann-Whitney U test. LAD = Left ascending coronary artery.

When statistical relationships between intestinal bacteria and different factors in CHD and non-CHD groups were analyzed, a correlation was found between age and the *Clostridium leptum* group (0.416, p = 0.001, Spearman’s rho). Furthermore, the *Clostridium leptum* group correlated with LAD stenosis (0.260, p-value = 0.035) and LAD calcification (0.351, p-value = 0.005). *Enterobactericeae* correlated with LAD fibrosis area percentage (0.442, p-value = 0.008), whereas *Bifidobacterium* spp. correlated negatively with LAD fibrosis area percentage (-0.411, p-value = 0.042).

### Bacterial DNA in coronary plaques

**Fig 2:**
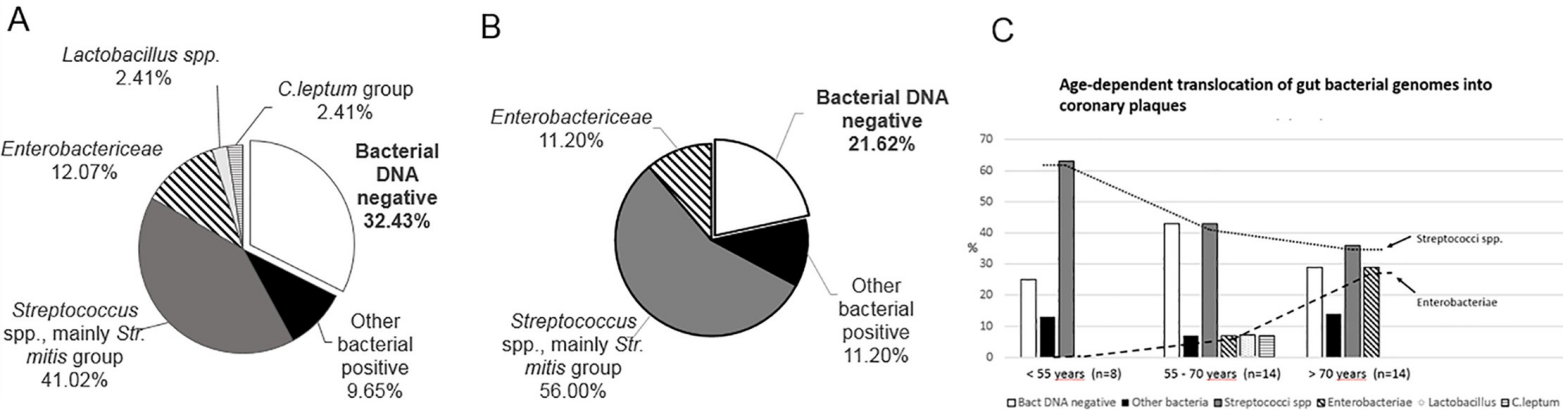
Gut bacterial translocation and bacterial DNA positivity in coronary plaques (n = 37). 2A. Bacterial DNA detected in coronary artery samples (total n = 37) using specific primers and probes for *Bacteroides* spp., *Clostridium (C*.*) leptum* group, *Clostridium coccoides* group, *Bifidobacterium* spp., *Enterobactericeae*, *Lactobacillus* spp., and *Streptococcus* spp. in all samples. The total amount of gut bacteria was measured using universal bacterial primers and probe described in Tuomisto et al., 2014 [25]. 2B. Bacterial DNA detected in coronary artery samples of CHD samples. 2C. Age-dependent translocation of gut bacteria into plaques.

Bacterial DNA was found in 68% of coronary samples, while 33% of coronaries did not contain any amplifiable bacterial DNA and were thus considered bacterial-DNA-negative (Fig 2A). DNA of the *Streptococcus* spp. (mainly of the *S*. *mitis* group) was the most common finding (41.02%), followed by *Enterobactericeae* (12.07%), the *Clostridium leptum* group (2.41%), and *Lactobacillus* spp. (2.41%). Based on the amount of bacterial DNA detected by universal primers, 9.65% of the coronary plaque samples carried DNA of bacteria not covered by the specific primers. When the analysis was restricted to CHD cases, the amount of *Streptococcus* spp.–positivity increased and the amount of bacterial-negativity decreased, but the results were not statistically significant (Fig 2B).

The number of bacteria-positive findings was found to increase with age, whereas bacteria-negative findings did not show any age-dependence (Fig 2C). The most important finding was the percentage of *Streptococcus* spp. DNA decreased and *Enterobactericeae* increased in coronary plaques with age, but these trends did not reach statistical significance, most probably due to the small number (n = 37) of cases.

The bacteria-positive group tended to suffer from more severe coronary artery disease, but differences were not statistically significant, possibly partly due to the small size (n = 37) of the study population. The bacteria-positive group had more severe percentages of coronary stenosis (43.5% vs. 32.3%. respectively, p = 0.212) compared to the bacteria-negative group. Correspondingly, the bacteria-positive group tended to have a larger fatty streak area (5.3% vs. 0.0%, p = 0.166), fibrosis plaque area (17.5% vs. 15.9%, p = 0.934), and calcification area (4.2% vs 3.8%, p = 0.704).

## Discussion

In this autopsy study, we utilized qPCR to measure age-dependent differences in the composition of major commensal gut microbiota and their association with coronary atherosclerosis in order to investigate the possible role of the gut microbiome in the development of heart disease. To our knowledge, we were the first to be able to measure age-dependent changes in gut bacteria and the presence of these bacteria DNA in coronary plaques in the same individuals. We have previously reported that fecal samples from autopsies can be reliably used up to 5 days post-mortem [23]. This is further supported by the present finding that the composition of the gut microbiome’s major populations did not differ between healthy volunteers and autopsy cases.

We found that the relative ratios of fecal *Lactobacillus* spp., *Bifidobacterium* spp., the *Clostridium coccoides* group, and *Bacteroides* spp. did not change with advancing age. In contrast, ratios of the *Clostridium leptum* group, *Enterobactericeae*, and *Streptococcus* spp. showed age-dependent increases. Subjects with more advanced coronary atherosclerosis were more likely to statistically significantly harbor *Clostridium leptum* group and *Enterobactericeae* DNA in their feces than those with less advanced atherosclerosis or healthy coronaries. We also detected bacterial DNA belonging to bacteria typical of the gastrointestinal tract in over half the coronary plaques, the most common being *Streptococcu*s spp., followed by *Enterobactericeae*, *Clostridium leptum*, and *Lactobacillus* spp. Roughly one third of plaques did not contain any bacterial DNA. The percentages of *Streptococcus* spp. -positive DNA findings decreased and *Enterobactericeae-*positive findings increased in coronary plaques with age. The hypothesis is that with advancing age, the intestinal epithelium becomes more permeable [34], enabling pathogenic bacteria to enter the portal vein and end up in circulating blood. It has been shown that T cells specific to microbial species can be found in large numbers in peripheral blood and are also abundant in atherosclerotic plaques [35]. Gut translocation of bacteria into blood and to distant organs has been proven in both animal and human studies [36]. However, we don´t absolutely know that the bacteria originated from the gut, but e.g., *Enterobactericeae*, *Clostridium* spp. and lactobacillus spp. are part of common intestinal microbiota and they have rarely or never been found in the oral cavity. However, we have also detected gut bacteria in liver samples of the same individuals suggesting that they may reach liver via the portal vein [25]. Another theoretical possibility for the route of bacterial translocation could be through anal fissures or bleeding hemorrhoids, which are common in old age. Bacteria could be translocated to coronary plaques most probably through neovascular channels developing inside a growing coronary atheroma. A further possibility is that ingested bacterial genomes are transferred into plaques inside macrophages participating in inflammation of coronary atheromas.

To date, research on the significance of composition of the intestinal microbiota in the development of atherosclerosis and CHD has been scarce. Previously it has been reported that CHD patients have increased amounts of *Prevotella* spp. and *Collinsella* spp., and decreased ratios of *Roseburia* spp. [5] and *Eubacterium* spp. [5] in their gut. Recently, it has been suggested that the composition of gut microbiota may affect the development of cardiovascular disease by producing atherogenic metabolites, such as trimethylamine (TMA), from dietary lipid phosphatidylcholine (lecithine), choline [37], or L-carnitine, which are present specifically in red meat [38].

We found that *Enterobactericeae*—Gram-negative bacteria producing LPS—were age-dependently increased in feces and associated with severe coronary disease, and were found in the coronary plaques of older individuals. There has been discussion on the role of lipopolysaccharides (LPS), found in the outer membrane of Gram-negative bacteria, in obesity and the development of heart disease. Moreira et al. [10] suggested that bacterial components, such as LPS, peptidoglycan and the lipoteichoic acids derived from the gut bacteria, are able to promote systemic low-grade inflammation, insulin resistance, and increased cardiovascular risk. LPS passes through the gut barrier from the lumen in chylomicrons first into the lymphatic system and then into blood. The higher the dietary fat content is, the more LPS are transported through the barrier [39]. LPS are carried further in the blood within lipoproteins, *e*.*g*. LDL. In the endothelium of blood vessels, LPS activate Toll-like receptor 4 (TLR4) receptors and macrophages to release pro-inflammatory cytokines, which may lead to endothelial dysfunction, plaque formation and rupture, as well as the oxidation of LDL and thrombogenesis.

Age is the most important determinant of CHD [40]. However, it is not completely understood how age inflicts its detrimental effect on coronaries. Older individuals have endured longer exposure to dyslipidemia and other CHD risk factors, such as hypertension and smoking. One possible age-dependent route of pathogenesis supported by our results is a change in the gut’s bacterial populations, leading to an increase in pathogenic bacterial populations [15, 16], as well as intestinal leakage of these bacteria under circumstances of a weakened immune defense in the gut epithelium [19].

In the present study, the relative amount of *Bifidobacterium* spp. correlated negatively with fibrosis percentage. This suggests that *Bifidobacterium* spp. might have a beneficial role in the prevention of coronary atherosclerosis. The administration of bifidobacteria has been shown to improve mucosal barrier function by preventing bacterial translocation and reducing the amount of LPS in blood [41].

It may be hypothesized that, as pathogenic bacteria increase in the gut, the possibility of translocation also increases and these pathogens can thus enter the circulation, ending up in coronary plaques. In line with our present results, phylotypes of *Streptococcus* spp. have previously been found in thrombus aspirates of patients with lower-limb thrombosis [30] and patients with myocardial infarction [31]. In support of our findings, Koren et al. also found that several bacterial phylotypes were present in both the atherosclerotic plaque and the gut, suggesting that bacteria present in the atherosclerotic plaque could have originated from the intestines [2].

Limitations of the present study include the fact that we have focused only on changes within specific major intestinal bacterial populations and we have no data on changes in the diversity of the entire gut microbiome. Since microbiota composition can be profoundly affected by several confounding factors, such as diet, which we were not able to control, the interpretation of these limited data should be conducted with caution. In particular, microbiome–drug interactions should definitely be taken into consideration, as many older individuals are administered antibiotics and other drugs. One limitation is that our study series comprised only males. Intestinal bacteria composition varies between males and females, and creates a source of variability. However, using only one gender, here males, this difference can be eliminated. Since we measured only bacterial DNA, we do not know whether our findings in the coronaries are due to the presence of whole live bacteria, or just bacterial DNA fragments brought by macrophages participating in inflammation of coronary plaques. The bacterial DNA was extracted from fecal samples with the Zymo Fecal DNA extraction kit and from coronary arteries with the Qiagen MiniKit. The only difference between the kits is that there is an extra step for DNA purification in the fecal procedure. Fecal samples are known to contain many inhibitors in contrast to coronary arteries. We believe this additional step in DNA extraction does not interfere with bacterial population detection.

## Conclusions

The present results suggest a possible direct role of gastrointestinal bacteria in the development of coronary heart disease but these observed associations may not infer causality and the result may be biased by confounding factors. More research on this topic is needed, however. In the future, there might be possibilities to prevent the development of coronary heart disease by modulating the content of intestinal bacterial populations.

## Supporting information

S1 Data Data for the study. (XLSX)
